# Influence of force volume indentation parameters and processing method in wood cell walls nanomechanical studies

**DOI:** 10.1038/s41598-021-84994-0

**Published:** 2021-03-11

**Authors:** Aubin C. Normand, Anne M. Charrier, Olivier Arnould, Aude L. Lereu

**Affiliations:** 1 Aix Marseille Univ, CNRS, CINaM, Marseille, France; 2 LMGC, Université de Montpellier, CNRS, Montpellier, France; 3 Aix Marseille Univ, CNRS, Centrale Marseille, Institut Fresnel, Marseille, France

**Keywords:** Atomic force microscopy, Plant sciences

## Abstract

Since the established correlations between mechanical properties of a piece of wood at the macroscopic scale and those of the cell wall at the submicron scale, techniques based on atomic force microscopy (AFM) have become widespread. In particular Peak Force tapping, allowing the differentiation of various layers, has become the new standard for wood cell wall’s nanomechanical characterization. However, its use requires fully elastic indentation, a good knowledge of stiffness of the probe and assumes a perfect tip shape of known radius (sphere) or angle (cone). Those strong hypotheses can result in large approximations in the extracted parameters for complex, nanostructured, and stiff and viscous materials such as wood. In this work, we propose a reliable and complementary alternative based on AFM force-volume indentation by refining the Oliver and Pharr nanoindentation processing and calibration procedure for AFM cantilever and tip. The introduced area-function calibration (AFC) method allows to considerably reduce these approximations and provides semi-quantitative measurements. No prior knowledge of the tip shape and cantilever stiffness are required and viscoplasticity is investigated through a qualitative index. Indentation parameters variations are shown to impact the resulting measurements, i.e., indentation modulus, viscoplasticity index, adhesion force and energy. AFC method, applied to map regions of tension wood, provides very stable mechanical parameters characteristic of each region, which makes this method of high interest for plant cell wall studies.

## Introduction

**Figure 1:**
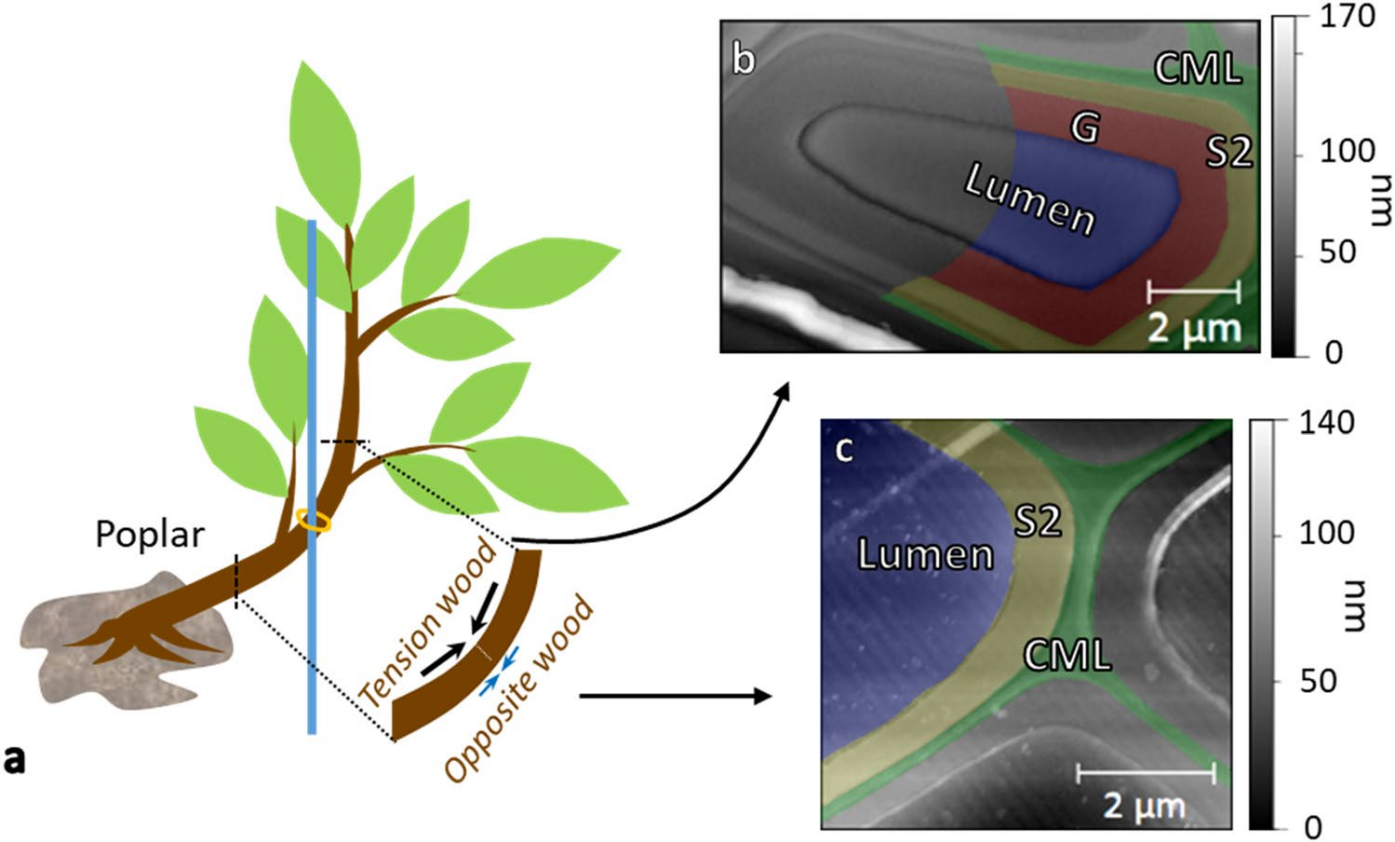
(**a**) Scheme of the tension generation in young poplar trees. (**b**, **c**) AFM topographic images of embedded and ultra-microtomed transverse cuts of tension wood fiber (**b**) and opposite wood fiber (**c**).

Wood mechanics has become an important field of investigation in correlation with the raising circular green economy and industry. Natural fibers are, for example, increasingly considered as an alternative to glass fibers in composite materials with polymer resins^1^. However, what prevents a generalization of the use of these materials is, among others, the wide variability of their mechanical properties. Variability at the scale of the work-piece is heavily influenced by the variability at the fiber scale^2^. Indeed, wood is a multi-scale material whose mechanical support function is ensured by the fiber’s walls. Wood cell walls can be seen as a multi-layered composite material in which partly crystalline cellulose microfibrils act as a unidirectional reinforcement. These microfibrils are oriented at a specific angle to the direction of the fiber (the microfibril angle, MFA) whereas the matrix mostly consists in amorphous lignin and hemicellulose^3–6^. In the so-called “normal” wood, the secondary layer (S2) (Fig. 1c), the thickest one, predominantly conditions the mechanical behavior of wood in the longitudinal direction. In tension wood, which is generated by angiosperms to reorient their axes through the generation of internal tensile stress during the maturation of new cells, sometimes a specific layer called “gelatinous” (G layer) (Fig. 1b) replaces or is added to the S2^7–10^. This layer is composed of a higher amount of crystalline cellulose almost aligned with the direction of the fiber (MFA close to 0°) and of  mostly hemicellulose for the matrix^7,8,10,11^. In addition to the industrial interest, knowledge of the mechanical properties of wood at parietal layers’ scale presents a fundamental biological interest for understanding the state of stress within the living tree, and, in particular, for providing information on how the G layer leads to the generation of maturation stress^8,9,12^. Different hypotheses exist to explain this phenomenon, but the lack of micro/nano-mechanical data does not allow to clearly understand how stresses and strains develop within each layer during maturation (especially the G layer) and how they are transferred from one layer to the other to explain the stress observed at the macro-scale.

Nanoindentation has been widely used to perform mechanical characterization in wood science since 1997^13^ at the cell wall scale. However, indentation leads to a complex mechanical stress state, consisting of compression in the direction of indentation and both compression and shear in the transverse direction. Furthermore, because the wood cell wall is not an isotropic material, indentation does not allow a direct determination of elastic constants of the tested material, but only provides an indentation modulus (M) resulting from the combination of these elastic constants, as it has been shown theoretically^14–16^. These theoretical works have already been applied to wood, allowing to estimate the elastic constants from successive indentation tests, and to deepen the understanding of the mechanical behavior of wood at the level of the cell wall^17^. Since traditional indenters do not allow resolving the thinnest parietal layers (like the compound middle lamella CML of the order of 100 nm), the use of atomic force microscope (AFM) has become widespread for carrying out nanomechanical tests on plant cell walls u*s*ing nanometric size tips as indenters. Mechanical properties mapping is an increasingly common feature in AFMs. Peak force quantitative nanoscale mechanical characterization (PF-QNM) or quantitative imaging (QI) present the advantage of reducing the volume of indented material, achieving excellent spatial resolution (indentation depth < 5 nm) and acquiring maps of mechanical properties at high speed^18–22^. These technics are based on an intermittent high frequency contact mode (>> 100 Hz) coupled to the choice of a model describing the mechanics of the contact between the tip and the indented material. The elastic indentation modulus is then extracted by adjusting its value to fit at best the force–displacement curves acquired with the chosen model. Various models, such as Hertz, JKR, and DMT, exist for simple tip geometries (i.e., conical, spherical), interacting with an indented volume considered as a flat half space. These models are only valid for contacts remaining in the elastic domain^23,24^, therefore, making impossible the study of viscoplastic behavior. Indentation depths associated with the PF-QNM mode are generally very small, very often less than the radius of the tip. However, because of surface roughness (due to the samples surface preparation) and of the small radius of curvature of the tips (necessary to reduce the contact area and to increase the resolution), the hypothesis of an elastic contact is not always verified. Thus, measurements can be biased if the contact regime is not perfectly elastic or if the shape of the tip is poorly determined. In addition, the extraction of the indentation modulus by this method is highly dependent on a precise determination of the cantilever stiffness, which is particularly difficult when it exceeds 20 N/m^25^, yet necessary to have a cantilever stiffness in the same order of magnitude as the indentation stiffness of wood cell walls. In order to limit these difficulties, it is recommended to determine beforehand the contact radius of the tip used on a reference sample with a known indentation modulus^18,21^ or with a calibration grid sample^26–28^ for a given indentation depth.

In the present work, we propose an alternative procedure, complementary to PF-QNM, based on AFM Force-Volume (FV) indentation mode with a data processing method adapted from the one traditionally used for nanoindentation^13,29–31^ coupled with a method of calibration of the tip that we named area-function calibration method (AFC) using a reference sample. Mechanical measurements made using FV mode and evaluated with Oliver and Pharr method have already been performed on several materials such as pulp fibers^32^ or metal nanosheets^33^ for example. They are highly affected by various parameters such as surface roughness (induced, among others, by the sample surface preparation process), cantilever calibration or the model used to describe the geometry of the tip. Our aim here is to refine this procedure by establishing a calibration and processing method to limit the source of potential errors and by studying the influence of indentation parameters on the calculated mechanical properties. Acquisition of indentation curves in FV mode is slower than in PF-QNM but offers the possibility of measuring the indentation modulus without requiring the hypothesis of perfectly elastic indentation. In addition, the AFC method makes it possible to determine the indentation modulus independently from the knowledge of the cantilever stiffness and also avoid the need to know the real shape of the tip beforehand, it therefore allows the use of atypical ones. It consists in building a tip/surface area function, i.e., the tip/sample contact area versus the indentation depth function, using a reference sample of known indentation modulus with a low surface roughness. Moreover, as the average indentation depth can be greater and the indentation times longer in FV indentation than in PF-QNM, the viscoplastic behavior of the material can be investigated. This is another attractive point of the presented method. We use a qualitative index characterizing the viscous and plastic behaviors of the indented material^23^, two properties of plant cell walls that are yet largely unknown at this scale. Furthermore, the adhesion is measured both as the force and the energy required to detach the tip from the indented surface. We focus on the comparison of these two computing methods and on the interpretation of such measurements applied to wood cell walls.

In order to validate our method, our protocol is applied on two types of parietal layers found in poplar: S2 layers from opposite wood (OW) cell walls and G layers from tension wood (TW) (Fig. 1). Two types of AFM probes are used to conclude on the influence of the tip. Finally, the influence of indentation time and depth on the determination of indentation modulus, viscoplasticity index and adhesion are investigated in order to choose the optimal parameters to establish correlative mappings of these properties.

## Results

### Brief overview of the method and extracted parameters

**Figure 2:**
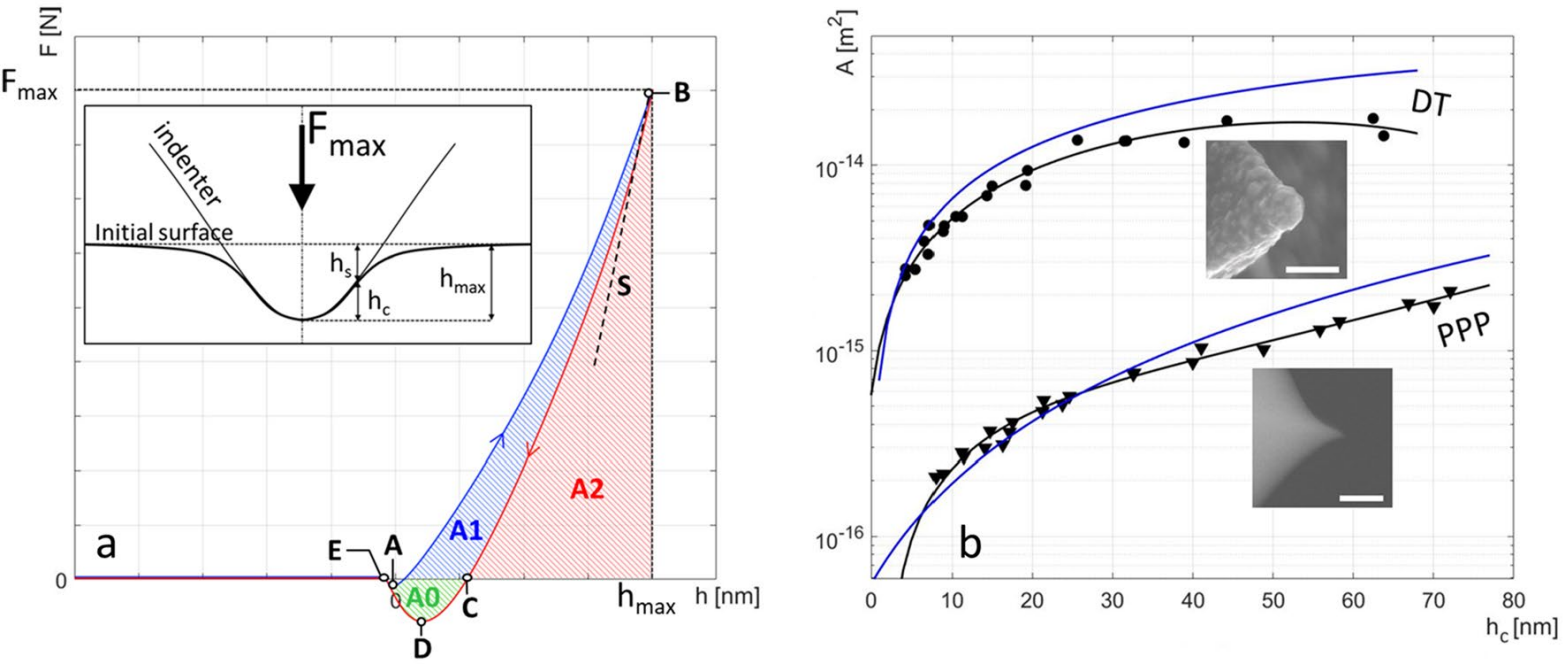
Overview of the indentation curve processing and area function calibration procedure. Inset: scheme of tip-surface interaction during indentation at maximum load. (**a**) Typical indentation measurement with loading (blue) and unloading curves (red). The hatched areas A0, A1 and A2, are used for characterization of adhesion and viscoplasticity respectively. (**b**) Contact area function for both DT and PPP probes as a function of indentation depth h_c_ using AFC method (data points-black symbols- and fits-black lines) and adjusted spherical-conical models (blue lines, Supplementary Section S1). Insets: Electronic microscopy of DT (scale bar = 400 nm) and PPP tips (scale bar = 100 nm).

Both AFC tip calibration and data processing methods are described in detail in the “Materials and methods” section. However, for a better reading of the manuscript a brief description is given in the following. Indentation curves are acquired using FV mode and processed with a dedicated Matlab program (see Fig. S1 and S2). A representation of such an indentation curve (loading and unloading curves in blue and red respectively) is shown in Fig. 2a with a scheme of the tip-surface interaction during indentation at maximum load (inset). The first step of the AFC method consists in establishing the area function, which describes the evolution of the tip/surface contact area with the indentation depth. Two examples, obtained using a reference sample of known elastic modulus, are given in Fig. 2b for two different tips (5 nm radius PPP probe and 110 nm radius, diamond coated, DT probe). We show that, using this area function, one can calculate directly the indentation modulus (M) after extraction of the slope ***S*** at the maximum of the unloading curve (Fig. 2a) without prior knowledge of the cantilever stiffness nor approximation on the tip shape. The proposed method including the extraction of ***S ***(discussed later), is an improvement of the popular Oliver and Pharr^30^ method for processing FV curves.

In addition to M, two other parameters are extracted and will be found useful to discuss the properties of wood samples later in the manuscript. The viscoplasticity index (VPI) is a qualitative index which represents the weight of the viscous and plastic components in the material response to indentation. This parameter, which is rarely exploited in AFM nanoindentation experiments, has been very useful to describe the viscoplastic properties at the nanoscale independently of the elastic properties. Our method is complementary to the method developed by Ganser et al.^34^ to acquire quantitative viscoelastic information using AFM nanoindentations. The later requires however very strong hypothesis (1. simple linear unidimensional stress–strain relationship, stress = strain * Young modulus, that is usually limited to uniaxial tests for isotropic material, 2. JKR-spherical contact, which implies to work in the elastic domain) and long measurements (600 s/measurement) that are not suitable for mapping. On the contrary, our approach consists in reducing the amount of hypothesis at the cost of having qualitative results rather than quantitative. The last parameter, adhesion, i.e., the force (F_adh_) or the energy (E_adh_) needed to separate the tip from the surface, reflects the interaction between the tip and the surface and can be used to deduce chemical information from the investigated sample. Although very common, this parameter has not been extensively investigated in the case of wood samples; we will show here how this parameter can be correlated to the chemical composition of each layers of the cell wall.

### Comparison of indentation modulus extracted with a geometric model or following the AFC method

To validate our data processing method, we carried out sets of indentation measurements in the G layer of the tension wood cell walls, using the two different probes, while varying the indentation depth. The number of indentations per set of measurement is limited by the width of the investigated layers (less than 1 µm). To avoid overlapping solicitated volumes, the number of indentations was limited to 3 × 3 grids replicated 3 times for a total of 27 indentations.

**Figure 3:**
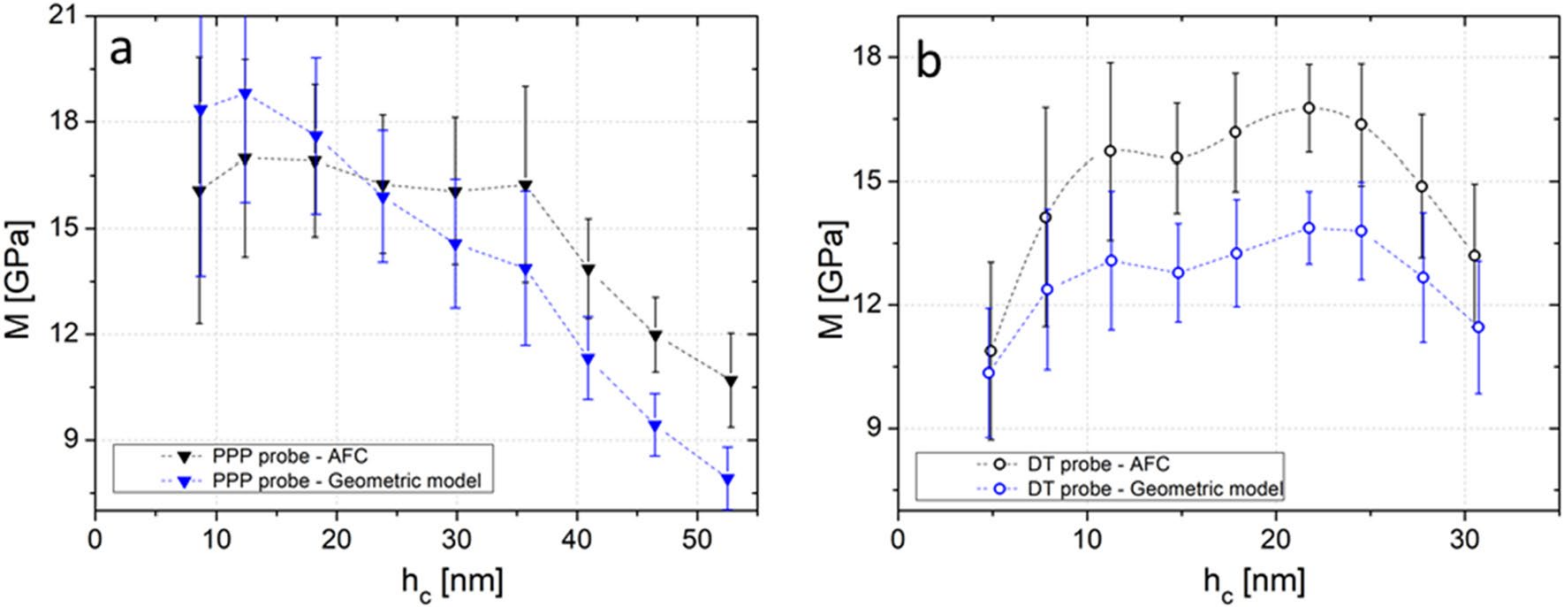
Indentation modulus as a function of indendation depth for: (**a**) PPP probe, (**b**) DT probe on tension wood G layer. Indentation modulus is computed using the area function calibration (AFC) (black) or using spherical-conical geometric model (blue). The dash-dotted lines are guide for the eye. Error bars are standard deviation, n = 27.

For both probe types (PPP and DT probes), indentations were performed and processed using our area-function calibration method described previously (example of raw indentation curves are available in Supplementary Section S1). We then compare our AFC method to a spherical-conical tip model to describe the probe geometry (Supplementary Section S1). This model, which is believed to be the most representative of the AFM tips used in our experiment, is a continuous model that considers a spherical shape at indentation depths smaller that the tip radius of curvature coupled with a conical shape for higher indentation depth. The indentation moduli extracted using the two methods are reported in Fig. 3, in black for the area function calibration method and in blue for the geometric model. Data obtained with the PPP and DT probes are shown in triangles and open circles respectively. The analysis was performed for values of the indentation depth *h*_*c*_ ranging from 5 to 30 nm for the DT probe and from 8 to 53 nm for the PPP probe. These two ranges of indentation depths are given by the experiment itself, the lowest values correspond to the limits for the contact detection and the highest ones to the cantilevers’ deflections linearity limits. For a given material, these ranges are hence cantilever and tip dependent.

The first observation is that the AFC method allows the extraction of the indentation modulus, here ~ 16 GPa, independently from the type of probe and indentation depth in a specific range. This range goes from 10 to 35 nm for the PPP probe and from 10 to 25 nm for the DT probe (black curves in Figs. 3a, b). Note that this value is in good agreement with previously reported data from the literature (15 GPa for poplar G layer^35^, 18–21 GPa for flax G layer^20^). Below the 10 nm limit, the modulus seems underestimated. Since the mechanical properties shall not depend on the depth of indentation in the parietal layer, this observation may be due to surface damaged by the cutting process or to surface roughness. Indeed for small indentation depths, the roughness has a great influence on the volume of material loaded resulting in an over-estimation of the contact area. For large indentation depths, i.e., h_c_ > 35 nm for the PPP tip and > 25 nm for the DT tip, the measured modulus drop could be explained by a change of indentation regime between the reference resin and wood cell walls, as well as microfibrils buckling under high loads^36–38^. Another explanation would be that the solicitated volume can overflow into less rigid regions, reducing the measured indentation modulus *M*. This effect was already highlighted^31,39^, with Berkovich nanoindentation for higher indentation depths (> 300 nm). It is therefore crucial before any FV measurements, to check for the adequate range for which the indentation modulus is independent from the indentation depth.

In contrast, the results of data processing using a geometric model show an underestimation of *M* with the DT tip (open blue circles, Fig. 3b), and a dependency of *M* with the indentation depth for the PPP probe ranging from 5 to 18 GPa for an indentation depth in the range 8 to 53 nm (blue triangles, Fig. 3a). This does not reflect the mechanical properties of the parietal layer, and illustrates the importance of the data analysis method in getting physically relevant data. The AFC method described here not only provides reliable and reproducible data, whatever the size of the tip apex, but also provides a way to use atypical tips with weird and unknown shapes^40^ or high roughness such as encountered with the DT tips (Inset Fig. 2b).

### Influence of indentation time and depth on the physical characteristics of wood cell wall

Using the AFC method, we extracted the different properties of the wood cell wall, i.e., the indentation modulus *M*, the viscoplasticity index *VPI* (see “Materials and methods” section), the adhesion force and adhesion energy in two regions of the cell walls corresponding to tension and opposite wood. The two kinds of probes previously introduced, i.e., the DT and PPP probes, are used to emphasize our AFC method regardless of the size and geometry of the tip. In the following we explore how indentation time *t* and depth *h*_*c*_ influence these parameters with respect to the investigated regions and probes.

#### Influence of indentation time

**Figure 4:**
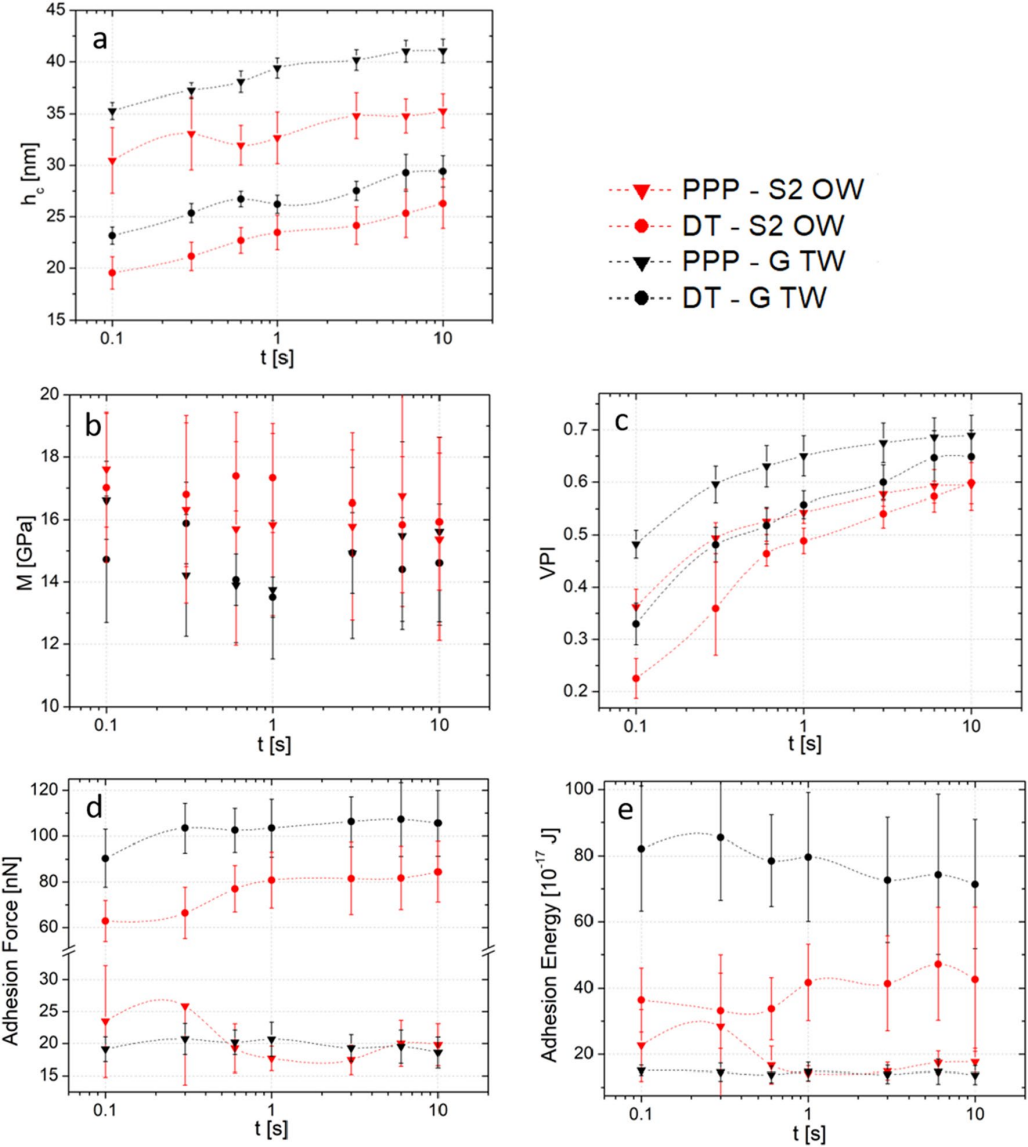
Influence of indentation time on: (**a**) indentation depth, (**b**) indentation modulus, (**c**) viscoplasticity index, (**d**) adhesion force, (**e**) adhesion energy. Measurements are made with DT probe (circles) or PPP probe (triangles) on S2 layer of opposite wood (OW, red) or G layer of tension wood (TW, black). The dash-dotted lines are guides for the eye. Error bars are standard deviation, n = 27.

In Fig. 4, we report the influence of indentation time corresponding to the time spent by the tip in contact with the indented surface during the loading and unloading of each test. This is not a parameter of primary importance for nanomechanical tests carried out by PF-QNM as the acquisition frequency in this mode is typically greater than 1 kHz. Creep is therefore negligible in such case. However, force-volume mode typically allows indentation time between 0.1 and 10 s and hence gives access qualitatively to time dependent properties of the wood cell wall.

In this study we investigated the effect of the indentation time *t* on the parameters described above (indentation modulus *M*, *VPI*, force and energy of adhesion) while keeping the cantilever vertical displacement identical for each measurement. We started by measuring the effect of *t* on the indentation depth *h*_*c*_ (Fig. 4a). In all cases we noted that the greater the indentation time, the greater the indentation depth. We observed an indentation depth variation by 15% for the PPP probe and 30% for the DT for an indentation time varying from 0.1 to 10 s, regardless of the tested cell wall region. This can be explained by the creep/relaxation of the polymer material under the tip with increasing time; when the indentation time goes up, the viscous component becomes more important in comparison to the instantaneous elastic component in the mechanical response of the material. This is confirmed by the viscoplasticity index (*VPI*), which is shown to increase as well with the indentation time (Fig. 4c). This demonstrates the relevance of this parameter to characterize the viscous behavior of the material. We note the *VPI* index is lower for the DT tip than for the PPP tip when looking at similar cell layers. This could be explained by the fact that strain and stress are comparatively lower for the larger DT tip than for the smaller PPP tip. Hence, probe-to-probe comparisons should not be done using the *VPI*, but it is very useful to compare qualitatively measurements performed with identical probes.

We also show that the indentation time has no significant effect on the measurement of the indentation modulus whatever the probe used (Fig. 4b). This result confirms that the AFC method, based on indentation modulus computation from the unloaded curve, makes it possible to overcome the effects of viscosity and plasticity, hence confirming that the measured modulus is an elastic modulus. It is also important to notice the large standard deviations (~ 2 GPa) in the indentation modulus. These deviations can be ascribed to two factors, first, to the error accumulated through the several steps of the process (sensitivity calibration, area function, indentation depth determination, slope computation), second, to the large volume being tested with FV experiments. Indeed, the great heterogeneity of the wood microstructure must be reflected in each of the indented volumes, which ultimately translates into the indentation modulus. In fact, the obtained standard deviations here are similar to those measured with other AFM modes or by nanoindentation in plant (mainly wood) cell walls^13,17–20,22,29,35^.

Finally, it appears that adhesion is not significantly influenced by the indentation time (Fig. 4d, e). However, adhesion measurement depends a lot on the probe used (e.g., shape, dimensions and roughness), and as for the *VPI*, comparisons of sets of data can only be made with identical probes. The variation between the two tips can be attributed to the difference in contact area or in chemical composition (See “Discussion” section).

#### Influence of indentation depth

Indentation depth is linked to the contact area, mechanical stresses and strains. It is therefore a fundamental parameter for understanding the mechanical response of the material. Force-volume mode allows exploring a wide range of indentation depths: from the minimal one, which corresponds to the limit of detection of the contact, up to cantilevers linearity limit. In this range, the indentation time also varies from 0.4 s (for a minimum indentation) up to 1.2 s (for a maximum indentation) due to the control of the cantilever’s displacement. However, comparison with results obtained while varying the indentation time makes it possible to isolate the influence of time and indentation depth.

**Figure 5:**
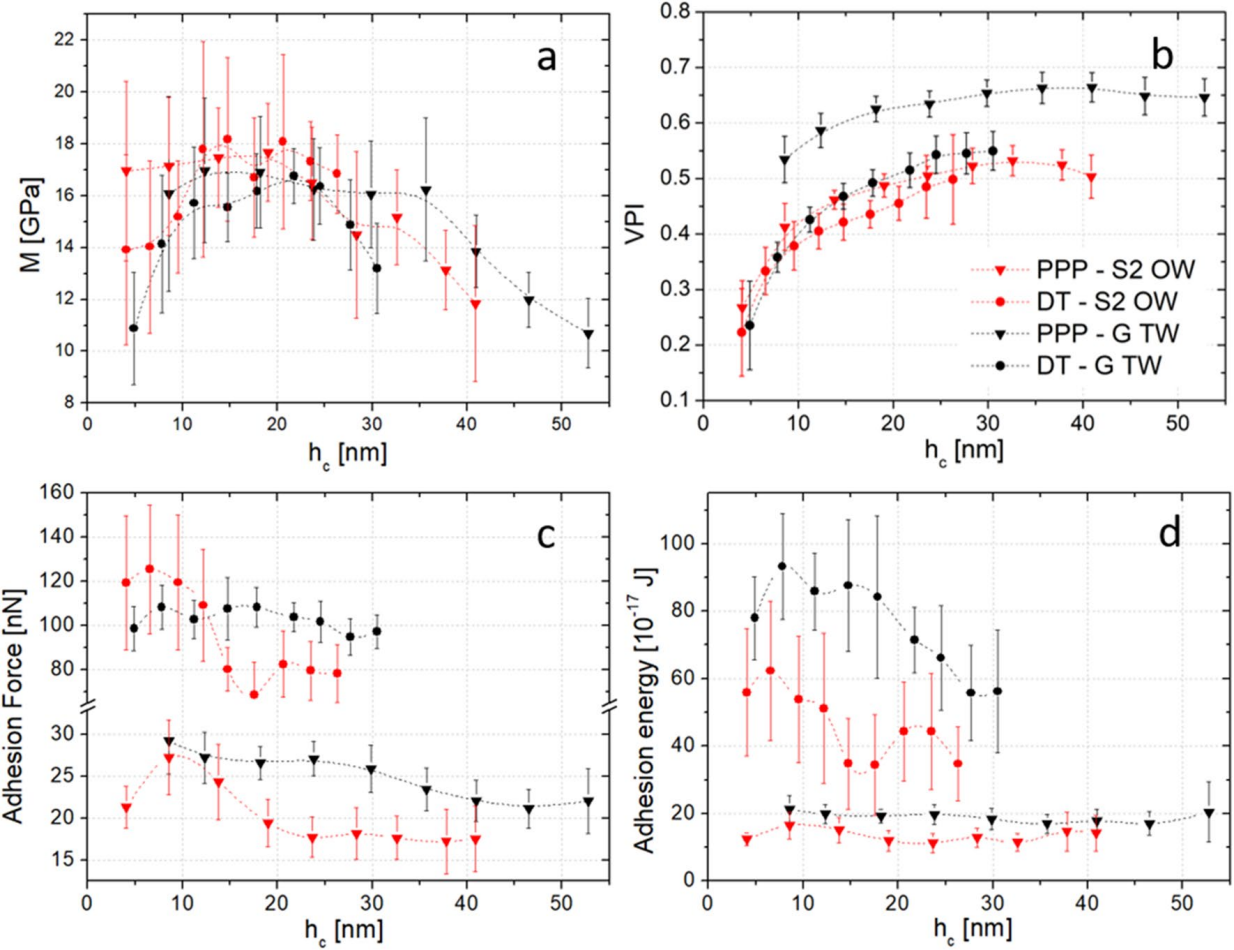
Influence of indentation depth on: (**a**) indentation modulus, (**b**) viscoplasticity index, (**c**) adhesion force, (**d**) adhesion energy. Measurements are made with DT probe (circles) or PPP probe (triangles) on S2 layer of opposite wood (OW, red) or G layer of tension wood (TW, black). The dash-dotted lines are guides for the eye. Error bars are standard deviation, n = 27.

As shown previously in the Comparison of indentation modulus extracted with a geometric model or following the AFC method section the indentation modulus exhibits a plateau, ranging between 10 and 35 nm for the PPP probe and between 10 and 25 nm for the DT probe, which corresponds to the adequate indentation depths to perform a correct measurement for a given tip (Fig. 5a). Below and beyond these limits, indentation modulus is underestimated, especially for the DT probe.

As expected, the viscoplasticity index increases with the indentation depth. For the PPP tip a plateau seems to be reached at *h*_*c*_ = 30 nm, while for DT the plateau seems to be reached at 25 nm (Fig. 5b). Note that the correct range of indentation depths cannot be determined universally and depends both on the shape of the probe and on the sample (surface roughness, microfibrils buckling, surface damage). *VPI* is lower with the DT tip than for the PPP, for the same layers and with comparable indentation depths. This reinforces our previous conclusion that *VPI* cannot be used to compare results obtained with two different probes. In addition, since *VPI* is not equal to zero, whatever the tip used, even for a very shallow indentation depth, we deduce that the indentation regime is never perfectly elastic. By comparing the results obtained by varying the indentation depth with those obtained by varying the indentation time, it can be seen that these two parameters both have an influence on this index. *VPI* is therefore the result of a convolution of the viscosity and plasticity through the effects of indentation time and depth. A small depth of indentation coupled with a long indentation time increases the preponderance of the viscous component in the *VPI* while a large depth of indentation coupled with a short indentation time makes it possible to highlight the plastic component. In conclusion, the *VPI* can be used to establish qualitative comparisons between indented samples for the same tip at the same depth and indentation time. Finally, indentation depth has no clear impact on the measurements of the adhesion force or energy in regard to the statistical dispersion (Figs. 5c, d).

## Discussion

### Impact of the indentation parameters in plant cell wall studies

By applying the recommendations and the method presented above, we explored the impact of the indentation parameters, depth and time rate, on the resulting wood cell wall analysis using two sets of parameters: The first set, set 1, combines parameters that are out of the recommended range, i.e., 2 nm indentation depth (far below the 10 nm minimal recommendation) and an indentation time of 0.02 s. The second set, set 2, follows the recommendations with an indentation depth and time of 10 nm and 0.7 s respectively. With these two sets, we performed two mappings of the same area at the intersection of three cells in tension wood using the PPP probe. An acquisition of the topography of the indented area was carried out between the two series in order to check that it was not damaged (Supplementary Fig. S3). Scanned area is 4 × 4 µm^2^, with a spatial resolution fixed at 50 nm (Supplementary Section S4).

**Figure 6:**
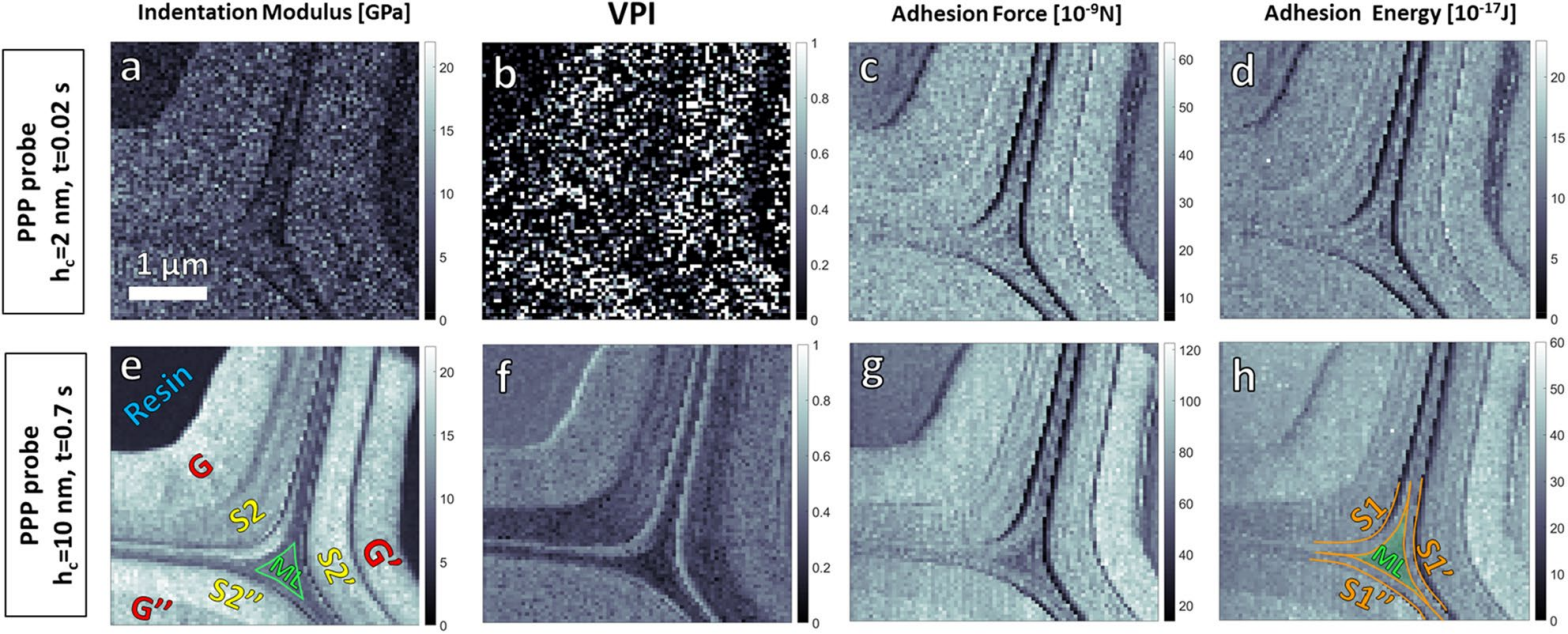
Maps obtained for two sets of parameters: (**a**–**d**) average indentation depth (*h*_*c*_) is 2 nm, indentation time is 0.02 s, (**e**–**h**) average indentation depth (*h*_*c*_) is 10 nm, indentation time is 0.7 s. (**a**, **e**) Indentation modulus, (**b**, **f**) viscoplasticity index, (**c**, **g**) adhesion force, (**d**, **h**) adhesion energy. Mappings are generated from sets of individual FV curves using a home-made Matlab software.

For each map, indentation modulus *M* (Figs. 6a, e), *VPI* (Figs. 6b, f), adhesion force (Figs. 6c, g) and adhesion energy (Figs. 6d, h) were extracted using the procedures described earlier. The first striking observation is that the mappings resulting from the two sets of indentations parameters differ drastically. As expected, in the case of small indentation depth, below the established recommendations, set 1, not only is the mapping of the indentation modulus very noisy and the moduli values are underestimated (around 12 ± 3 GPa for G), but it is also difficult to differentiate between the different layers of the cell wall (Fig. 6a). This contrasts drastically with the set 2 mapping where each layer (G, S2, S1 and compound middle lamellae CML) are clearly identified with a range of moduli that will be discussed further. Also using set 1 parameters, it turned out that force-volume mode reached its limits, probably due to a lack of control of the AFM regulation loop causing the retract curve to shift above the forward curve. *VPI* was therefore not determinable using set 1, and the resulting values are unusable (Fig. 6b). Although adhesion measurements seem less impacted by the choice of indentation parameters, (Figs. 6c, d, g, h), set 2 clearly allows resolving the different layers, in particular, S1, ML and even sometimes the primary wall, S1 can be distinguished. These results hence illustrate the importance of the parameters selected for the indentations measurements, and how these parameters can impact the results of the analysis.

Several information can be drawn from the mappings with the optimum parameters (Set 2) (indentation curves in Supplementary Fig. S4):

- Large variations at the transition between the different layers (resin, G, S1, S2, CML) can be explained by the high roughness in these areas due to cutting effects during sample preparation. These areas do not bring significant information and are therefore excluded from the analysis (Figs. 6e–h).
- Indentation modulus in the G layer seems constant whatever the portion of the cell observed, and close to 17 GPa (Fig. 6e) in agreements with other reported values^35^. In contrast, the indentation moduli in the S2 and S1 layers vary significantly depending on the portion of the cell observed (14–18 GPa for S2 and 9 to 14 GPa for S1) this difference can be explained by a variation of the microfibrils angle (MFA) at the surface generated by a cutting angle not perfectly perpendicular to the fiber angle as described in^35,41^. Note that the indentation moduli in the S2 layer is sometimes higher than those obtained in the G layer (see Fig. 4b) although due to the lower MFA in the G layer, one can expect it to have the highest value. In addition to the sample misorientation mentioned above, this difference could be explained here by the fact that: the indentation modulus is not very sensitive to the MFA when its value is low (i.e., < 15°)^4,42^ and/or buckling of the microfibrils^36^ could occur more easily in the G layer as its cell wall matrix is mainly composed of a hemicellulose gel-like structure^7,8,12^.
- *VPI* values present a low dispersion and allow a good discrimination of each layer, therefore it is a great physical property to look at in addition to indentation modulus. However, *VPI* in S2 varies greatly according to the observed area of the cell (Fig. 6f), even though there is no obvious correlation between the variations of modulus in S2 and the variations of *VPI*. We mainly observe that, for the two upper cells of Fig. 6, *VPI*_G_ ≥ *VPI*_S2,_ *VPI*_S1_ > *VPI*_CML_, the viscoplastic component hence contributes more largely to the mechanical response in G suggesting a higher viscosity or weaker plastic properties (lower elastic limit or greater plastic strain) than in other layers. In the case of negligible contribution of plasticity, these results are in agreement with damping factor (tan δ) mapping obtained by CR-AFM on chestnut TW at much larger loading frequencies^35^. They also partially agree with macroscopic viscoelastic dynamic characterization (DMA) of tension wood versus normal wood of tropical species^43^ at similar loading frequencies, where viscosity of TW was found higher than of OW's (or normal wood). Finally, even if *VPI* cannot be directly associated to creep behavior because of the plasticity contribution, our results are in agreement with recent studies using Berkovich nanoindentation creep test studies^44^ in which viscosity in normal wood was found higher in S2 than in CML.
- The way adhesion is measured, as a force (Fig. 6g) or energy (Fig. 6h), does not provide different biological information concerning the cell walls. This agrees with the DMT and JKR theories of mechanical contact, which take adhesion into account, and which indicate that the adhesion force is proportional to the adhesion energy per unit area^23^. Since the contact area is not known during the adhesion phase, it is impossible to know precisely the proportional factor between these two quantities. However, it can be seen that the ratio of the adhesion force to the adhesion energy is almost constant, whatever the location on the wood cell wall (between 2.3 and 2.5). Thus, whatever the parameter (force or energy) used to study adhesion, we observe a clear trend such as Adh_G_ > Adh_S2_ > Adh_S1_ > Adh_CML_ (Figs. 6g, h), with little variations within the S2 layer, and therefore little dependence to the MFA. This result hence suggests that adhesion could be sensitive to the chemistry of the different parietal layers. This hypothesis is supported by the existing correlation with the chemical composition of the matrix of each of these layers: the ratio of the (amorphous) hemicellulose (hydrophilic)/lignin (hydrophobic) fraction is more important within the G layer (no lignin, matrix made of hemicellulose in a gel-like structure^12^) than in S2, which is itself more important than in the CML^45^. Our measurements are in adequacy with the hypothesis that adhesion in ambient conditions reflects the affinity of the indented surface with water: a stronger adhesion corresponds to a more hydrophilic material. In the case of wood cell walls, this can be interpreted as an area rich in (amorphous) hemicellulose (cellulose molecules are rich in hydroxyls groups that give them hydrophilic properties), especially in the case of the G layer with a gel-like structure^8,12^. The local roughness, of the surface and of the tip apex, which is also known for having a huge impact on adhesion measurements^46^, is estimated from a topographic map of the area acquired using the AFM in semi-contact mode. Average roughness (R_a_) which ranges between 1 and 2 nm for G, S2 and CML shows no significant differences between these layers (Table 1), and does not correlate with the measured adhesion, hence strengthening the hypothesis of an adhesion characterizing surface chemistry through its hydrophilic properties. Table 1 summarized the various extracted parameters and the above comparisons are illustrated by the average values evidenced in bold.

**Table 1:** Physical parameters extracted from Figs. 6 e–h mappings for the different wood cell wall layers of three nearby wood cells. n is the number of indentation measurements performed for each analyzed region.

	S_a_ (nm)	M (GPa)		VPI		F_adh_ (nN)		E_adh_ (e^−17^ J)		F_adh_/E_adh_ (m^−1^)	n
G	1.5	17.3 ± 1.6		0.50 ± 0.06		91.0 ± 7.1		39.1 ± 3.3		2.3	889
G′	1.5	16.7 ± 1.4		0.45 ± 0.05		101.6 ± 5.9		44.5 ± 3.4		2.3	719
G″	1.2	16.4 ± 1.3		0.47 ± 0.03		90.5 ± 6.9		37.8 ± 3.7		2.4	185
G_ave_	**1.4**	**16.8**		**0.47**		**94.4**		**40.5**		**2.3**	
S2	1.9	13.8 ± 1.4		0.35 ± 0.08		84.2 ± 7.5		34.3 ± 3.4		2.5	860
S2′	1.9	17.2 ± 1.6		0.31 ± 0.06		89.0 ± 7.4		36.3 ± 3.5		2.5	609
S2″	1.3	18.2 ± 1.6		0.48 ± 0.05		87.9 ± 6.6		35.4 ± 3.5		2.5	386
S2_ave_	**1.7**	**16.4**		**0.38**		**87.0**		**35.3**		**2.5**	
S1	3.5	14.1 ± 3.6		0.36 ± 0.08		76.1 ± 7.6		31.7 ± 2.9		2.4	145
S1′	4.1	9.6 ± 1.9		0.32 ± 0.07		68.4 ± 10.8		31.5 ± 3.2		2.2	115
S1″	3.0	10.3 ± 0.9		0.27 ± 0.06		75.5 ± 8.1		32.0 ± 3.2		2.4	129
S1_ave_	**3.5**	**11.3**		**0.32**		**73.3**		**31.7**		**2.4**	
CML	**1.3**	**9.9** ± 1.2		**0.22** ± 0.05		**63.4** ± 7.4		**27.1** ± 2.6		**2.3**	75
RESIN	1.1	3.9 ± 1.8		0.43 ± 0.03		64.1 ± 3.6		37.7 ± 2.6		1.7	659

## Concluding remarks

The presented Area Function Calibration (AFC) method, allows to considerably reduce the approximations on materials elastic modulus resulting from a bad knowledge of tip shape and cantilever stiffness usually encountered with AFM force-volume measurements and open the path to semi-quantitative analysis. This reveals to be particularly useful to study (relatively) stiff materials, such as wood cell walls or other inorganic materials, requiring the use of cantilevers with a high stiffness, which is difficult to precisely determine. In addition, it opens the possibility for using probes not usually dedicated to indentation, such as diamond coated probes with high surface roughness or atypical probes with weird shapes.

Application of the method to an indentation mapping over a tension wood region allowed the quantitative mappings of the indentation moduli. It was established that the viscoplasticity index (VPI) depends both on the viscous and plastic behaviors of the indented material and turned out to be a stable and significant parameter complementary to elastic indentation modulus. Finally, adhesion was found to reflect the cellulose content through hydrophilicity and could be used as a complementary approach to other chemical mapping techniques (such as RAMAN spectroscopy) which do not allow such great spatial resolution. Thus, force-volume indentation with the presented method has proven to be a rich toolbox to study various physical, chemical properties and to carry out correlations to deepen our knowledge of wood cell walls.

We believe the AFC method brings the use of AFM force-volume mode to a higher standard where the extracted indentation moduli are semi-quantitative and we are convinced that it will be very useful to anyone interested in the nano-mechanical properties of complex materials.

## Materials and methods

### Sample preparation

Wood samples come from young 7-month-old poplars (*Populus euramericana*, “Robusta” and “I214” blend) cultivated in open-field and constrained with a tutor after two months of growth to induce tension wood generation (Fig. 1a). After harvesting, the samples are cut, stored in water and kept in a cold room at 5 °C.

To confirm the presence of tension wood, a saffranine-Alcyan blue staining was first performed on an anatomical section in order to reveal the presence of the cellulose rich areas corresponding to tension wood^47,48^.

Surface roughness is a critical parameter when it comes to AFM indentation measurements. As a result, the great challenge in sample preparation is to reduce this roughness as much as possible. Our procedure is the following: sample preparation starts with a 1 cm thick stems sections cut with a saw. Matches of an approximate size of 4 × 1 × 1 mm^3^ oriented according to the direction of the fibers are then extracted within the tension wood and the opposite wood. The matches are then dehydrated under vacuum in successive ethanol/distilled water baths with ethanol concentrations of 50%, 75% and 100% for half an hour each. Dehydration is necessary to allow inclusion in hydrophobic resin LR white (EMS Catalog LR White Medium grade), which is itself necessary to avoid the collapse of the gelatinous layer of tension wood^48^. Ethanol has been shown to be the solvent which minimizes the damages to the G layer^49^. LR-White has been chosen as the embedding resin that has reduced penetration into wood cell wall, leading to no change in mechanical measurements at the nanometer scale^50^. Resin inclusion is then obtained by soaking the matches overnight in a 1:1 Ethanol:LR white mixture at room temperature, followed by two successive resin baths for 1 h and 2 h respectively. Subsequent polymerization is carried out in an oven at 65 °C for about 24 h.

Finally, the extremity of the samples is cut into a pyramidal shape using a razor blade in order to reduce the section before ultra-microtoming. Ultra-microtomy (Leica EM UC7) is performed as best as possible perpendicularly to the axis of the fibers. The surface is leveled and the next 100 µm cut with a glass knife. Finishing cuts are carried out using an ultra diamond knife (RMC Ultra) (final cuts of 50 nm in thickness at 0.6 mm/s). With such procedure, the local roughness (R_a_), computed from topographic images produced in semi-contact mode by AFM (Figs. 1b, c) on the largest surfaces available of each layer was found to be smaller than 2 nm for all layers.

### AFM and tips

Topographic images were first acquired using a NTEGRA AFM from NT-MDT in semi-contact mode. Indentations were then performed using the same AFM in contact mode, by controlling the displacement of the piezoelectric element in the direction perpendicular to the surface and the time rate required to perform this displacement. All the tests were carried out below the limit of linearity of each cantilever which was previously determined.

Two types of tips were used: PPP-NCHR (PPP) from Nanosensors with a radius of curvature of 5 nm as assessed by scanning electron microscopy (SEM) measurements and a half-cone angle of 20° (manufacturer's data). We verified, using the area-function, that the shape of the tip remained unchanged after measurements. The calibration of this tip using the Sader's method gives a stiffness of the cantilever of 22 N/m. For the tip, made of silicon without any coating, elastic modulus and Poisson’s ratio of silicon are used as mechanical constants of the tip, i.e., E_Si_ = 180 GPa and ν_Si_ = 0.28 respectively^51^. The second kind of tips, the DT-NCLR from Nanosensor (DT), is not usually used for nanoindentation tests because of its irregular surface caused by a diamond coating to make it harder and limit its degradation. Its radius of curvature is estimated to 110 nm and presents a high surface roughness of a few nanometers. The half cone angle is 20° and the stiffness of the cantilever is estimated at 70 N/m after calibration by the manufacturer. The values taken as mechanical constants for the diamond are: E_diamond_ = 1200 GPa and ν_diamond_ = 0.2^52^.

### Determination of tip/surface contact area-function

The first step of our method consists in establishing the area function, which describes the evolution of the tip/surface contact area with the indentation depth. To build this curve several series of indentations were carried out on a reference sample with known indentation modulus at various indentation depths. For each measurement, the slope S at the beginning of the unloading curve can be expressed by^53^:

1 $$S_{0} \left( {h_{max} } \right) = \frac{{dF_{0} }}{{dh_{0} }}\left( {h_{max} } \right) = \frac{2}{\surd \pi } \cdot M_{0} \cdot \sqrt {A\left( {h_{c} } \right)}$$

where the “0” index refers to the reference sample, with *M* the indentation modulus, *F* the maximum force applied by the tip on the surface, *h*_*max*_ the total indentation depth and *h*_*c*_ the effective indentation corresponding to the contact depth (Fig. 2a)^29,30^.

Considering the cantilever as a linear spring, the force can be written as $F = k \cdot \delta$ with *k* and $\delta$ the cantilever’s stiffness and deflection, respectively. Let us note:

2 $$S^{\prime} = \frac{d\delta }{{dh}} = \frac{S}{k}$$

Using Eqs. (1) and (2), the area function can be expressed as a function of the indentation depth as:

3 $$\sqrt {A\left( {h_{c} } \right)} = \frac{\surd \pi }{2} \cdot k \cdot S^{\prime}_{0} \left( {h_{max} } \right) \cdot \frac{1}{M}_{0}$$

with

4 $$\frac{1}{{M_{0} }} = \frac{1}{{M_{Resin} }} + \frac{{1 - \nu_{tip}^{2} }}{{E_{tip} }}$$

where *E*_*tip*_ and *ν*_*tip*_ are the tip elastic Young’s modulus and Poisson ratio. The resin sample, assumed homogeneous, and calibrated in reference 18 by nanoindentation is used as reference, with *M*_*Resin*_ = 4.5 GPa^18,50^. Note that the reference sample must have an indentation modulus in the same range as those of the sample under investigation.

For each indentation curve, the four-steps processing goes as follows:

1. Conversion from deflection curves as a function of the displacement of the piezoelectric element to force curves as a function of the depth of indentation using the cantilever sensitivity (cantilever displacement versus deflection) measured beforehand on a sample considered to be infinitely rigid (here flat Silicon). Cantilever stiffness values provided by the manufacturer or obtained using Sader’s method^54^. It is shown here that this value does not impact indentation modulus determination.
2. Smoothing using a Savitsky–Golay function to overcome measurement noise.
3. Subtraction of the force baseline.
4. Determination of the slope S_0_ at the beginning of the unloading curve to reduce the effects of viscosity and plasticity.

Extraction of S_0_ is critical and may influence drastically the resulting values for the elastic modulus. The methodology that we have set to extract this slope is discussed in the following. Oliver and Pharr^24,30^ recommends fitting the unloading curve with a power law of the form $F = \alpha \cdot h^{m}$ , the slope is then computed as the derivative of this function at the beginning of the curve. In practice, unloading curves rarely follow such a function in the case of polymers^24^. To overcome this difficulty, S is sometimes approximated by a linear regression, however, the range chosen to fit the unloading curve greatly influence S’s determination^24^. We choose here a compromise between the two methods: the entire unloading curve is adjusted with a *m*th degree polynomial and we determine S as the maximum value of the derivative, i.e. corresponding to the slope at the beginning of the curve (Supplementary Fig. S5a). In supplementary section S5, values of S are computed using polynomials of degrees ranging from 1 to 8, each point corresponding to an average of 16 curves (Supplementary Fig. S5b). A polynomial of degree 5 is the best compromise between a good fit quality and a low influence of measurement noise.

To take into account the phenomenon of surface deflection during indentation (Fig. 2a), we assume a “sink-in” indentation behavior in both resin and plant cell walls^24,39^. Indentation depth is corrected using Oliver and Pharr formula (Eq. 5) with:

5 $$h_{c} = h_{max} - h_{s} ,\quad {\text{with}}\quad h_{s} = \varepsilon \frac{{F_{max} }}{S} = \varepsilon \frac{{k \cdot \delta_{max} }}{{k \cdot S^{\prime}}} = \varepsilon \frac{{\delta_{max} }}{{S^{\prime}}}$$

where the epsilon coefficient (*ε*) is introduced to take the indenter geometry into account.

It was shown that *ε* equals 0.72 for a conical shape, 0.75 for a parabola of revolution and 1 for a cylindrical flat punch^24,30^. However, because the shape of the tip is not always well known or does not match the simple geometrical models, we investigated the effect of the value of *ε* on the resulting indentation moduli. We show that these moduli have a relative variation of 10% at most for extreme values of *ε* ranging from 0.5 to 1, thus showing that this parameter has little influence on the final determination of the moduli (Supplementary Fig. S6). In this study we chose a value of *ε* = 0.75 for a parabola of revolution, the closest geometry of the tips we used.

Using Eqs. (3) and (5), we plotted, for each indentation depth *h*_*c*_, the tip/surface contact area *A* as shown in Fig. 2b for the two types of tips (See Methods section). For each tip, the area function, expressed as *A* = *f*(*h*_*c*_) is obtained by fitting the curves by a degree 3 polynomial function (Fig. 2b, black lines).

### Plant cell wall indentation data processing and extraction of indentation modulus

The indentation curves on the plant walls are processed in a similar manner to those of the reference sample described above. In addition to the 4 steps described previously, the last step consists in determining the remarkable points that are required to extract the different physical quantities (indentation modulus, adhesion force and energy, viscoplasticity index): contact point (A), start of the unloading curve (B), end of the indentation (C), maximum adhesion (D), end of the adhesion (E) as pointed in Fig. 2a.

As for Eq. (1), and using the area function determined previously with the reference sample, we can write the indentation modulus as:

6 $$M = \frac{\surd \pi }{2} \cdot S\left( {h_{max} } \right) \cdot \frac{1}{{\surd A\left( {h_{c} } \right)}}$$

By developing *A* and *S* from Eqs. (2) and (3), the sample modulus can be expressed independently of the cantilever stiffness as:

7 $$M = \frac{{\frac{\surd \pi }{2} \cdot k \cdot S^{\prime}\left( {h_{max} } \right)}}{{\frac{\surd \pi }{2} \cdot k \cdot S^{\prime}_{0} \left( {h_{max} } \right) \cdot \frac{1}{{M_{0} }}}} = \frac{{S^{\prime}\left( {h_{max} } \right)}}{{S^{\prime}_{0} \left( {h_{max} } \right)}} \cdot M_{0}$$

Since the plant cell walls are strongly anisotropic materials (transverse isotropic with axis of symmetry along the microfibrils direction) and that indentation test produces a complex loading, essentially composed of compression in the direction of indentation and compression and shear in the transverse direction, we cannot directly extract a Young's modulus, e.g., the longitudinal or transverse modulus^17,29,55^. Indentation modulus results from a combination of the elastic constants^4,15,16,56^, and is expressed as follows to take tip deformation into account^24,30^:

8 $$\frac{1}{M} = { }\frac{1}{{M_{wood\;cell\;wall} }} + \frac{{1 - \nu_{tip}^{2} }}{{E_{tip} }}$$

thus

9 $$M_{wood\;cell\;wall} = \left( {\frac{1}{M} - \frac{{1 - \nu_{tip}^{2} }}{{E_{tip} }}} \right)^{ - 1}$$

### Viscoplasticity index mapping

To deepen our knowledge on the mechanical behavior of plant fibers, we use the dimensionless qualitative viscoplasticity index (*VPI*) defined as: *VPI* = 1 − A2/(A1 + A2) (Fig. 2a)^23,57,58^. The area under the loading curve, A1 + A2, corresponds to the energy required to perform the indentation and under the unloading curve, A2, to the energy restored during the indenter withdrawal by the reversible relaxation of the sample. A *VPI* of 0 corresponds to a perfectly elastic indentation while an index of 1 corresponds to a perfectly plastic indentation.

### Adhesion force and energy mappings

Adhesion reflects the interaction between the tip and the sample surface. For measurements performed in air, at ambient conditions and with stiff sample as those here, adhesion has been shown to characterize the chemistry through the surface water content^59^. A water bridge forms between the tip and the indented surface, and the resulting capillary forces are predominant in the measured adhesion. However, adhesion shall be compared as a qualitative parameter as many authors have warned about the influence of surface roughness, tip chemistry and geometry^23,46^. Adhesion can be measured both as the maximum force of attraction during the separation of the tip with the surface or as an energy (*A*_0_) corresponding to the energy preventing the tip to detach from the sample during tip withdrawal (Fig. 2a).

### Measurements protocol and statistical indicators

Since the walls of wooden cells are comparable to a transverse isotropic material along the microfibrils direction, the angle of the microfibrils on the surface is a parameter of paramount importance influencing the value of the measured indentation modulus and associated mechanical parameters. An indentation carried out along the axis of the microfibrils results in the determination of a larger indentation modulus than for an indentation performed given a different angle^29,42,55^. The surface microfibrils angle depends both on the natural wood cell walls microfibrils angle with respect to the direction of the fiber (biological data, MFA), as well as the cutting angle (sample preparation dependence). This latter causes a variation of the microfibrils angle at the surface as a function of the indentation position around the cell^35,41^.

Although the purpose of the accurate cut is to be perpendicular to the direction of the natural fibers as best as possible, a small variation can have a large impact on the measured modulus. In order to limit this variability or to evidence the cutting angle impact, we acquired each set of indentations by replicating it 3 times at different positions around the same cell, or a neighboring cell.

Each set corresponds to 9 indentations arranged in a 3 × 3 grid with a gap between each indentation in order to avoid overlapping imprints and solicited volumes (Supplementary Fig. S7). The results of the 3 sets of 9 indentations made with the same parameters are then averaged and the error bars correspond to the standard deviation over these 27 measurements.

## Supplementary Information

Supplementary Information.

## Data Availability

The datasets used and/or analyzed during the current study are available from the corresponding author on reasonable request.
