# Supplementary material for: Influence of force volume indentation parameters and processing method in wood cell walls nanomechanical studies
Source: Sci Rep. 2021 Mar 11;11:5739. doi: 10.1038/s41598-021-84994-0 (PMC7970955; doi:10.1038/s41598-021-84994-0)
Supplement: Supplementary file 1 — Supplementary Information. [file 41598_2021_84994_MOESM1_ESM.docx]

# Influence of force volume indentation parameters and processing method in wood cell walls nanomechanical studies

Aubin C. Normand^1^, Anne M. Charrier^1^, Olivier Arnould^2^, and Aude L. Lereu^3*^

^1^Aix Marseille Univ, CNRS, CINaM, Marseille, France

1. LMGC, Université de Montpellier, CNRS, Montpellier, France
2. Aix Marseille Univ, CNRS, Centrale Marseille, Institut Fresnel, Marseille, France

*aude.lereu@fresnel.fr

# Supplementary materials

### **CONTENT**

1. Contact area function for spherical-conical model
2. Raw and processed indentation curves for PPP and DT probes on G layer
3. Surface control before and after indentation mappings with different parameters
4. Determination of maximum resolution
5. Typical Force-indentation depth curves for several layers of tension wood
6. Choice of polynomial degree for slope computation
7. Choice of the tip shape parameter Ɛ
8. Example of sets of indentation measurements in the S2 layer

## **1- Contact area function for spherical-conical model**

Supplementary equation (S1) for spherical-conical model : h indentation depth; R sphere radius; $\alpha$ half cone angle; A contact area

For an indentation depth (h) under sphere-cone tangent point : if $h\leq R(1-Sin \alpha)$

$$A= \pi(2Rh -h^{2})$$

For an indentation depth (h) above the sphere-cone tangent point : if $h > R(1-Sin \alpha)$

$$A = \pi{[Tan \alpha(a + h)]}^{2} with a = R ( 1 / Sin \alpha- 1 )$$

## **2- Raw and processed indentation curves for PPP and DT probes on G layer**

*
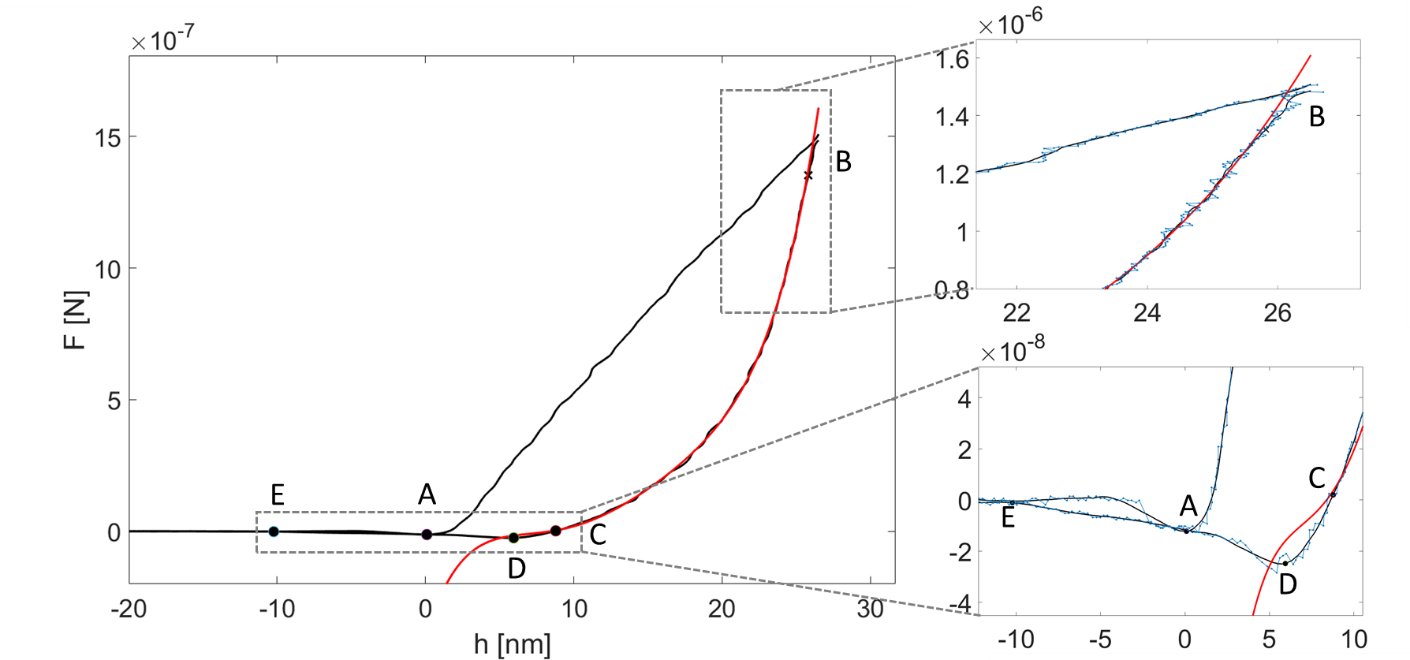
*

Supplementary figure S1: Force-Indentation depth curve on tension wood G layer using PPP probe, with raw data before smoothing (blue), after smoothing (black curve), degree 5 polynomial fit (red) and points of interest as presented in Fig. 2a. The zoom in Figs. S1b and c are given to evidence noise and adhesion, respectively.

*
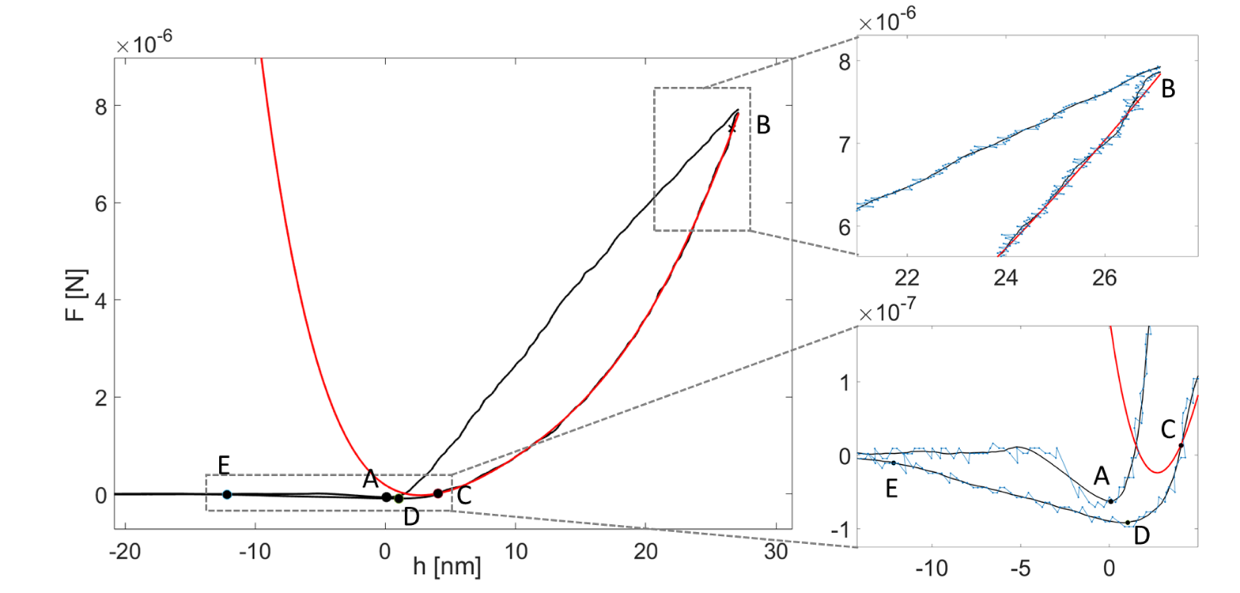
*

Supplementary figure S2: Force-Indentation depth curve on tension wood G layer using DT probe, with raw data before smoothing (blue), after smoothing (black curve), polynomial fit of degree 5 (red) and points of interest as presented in Fig. 2a. The zoom in Figs. S2b and c are given to evidence noise and adhesion, respectively.

## **3- Surface control before and after indentation mappings with different parameters**


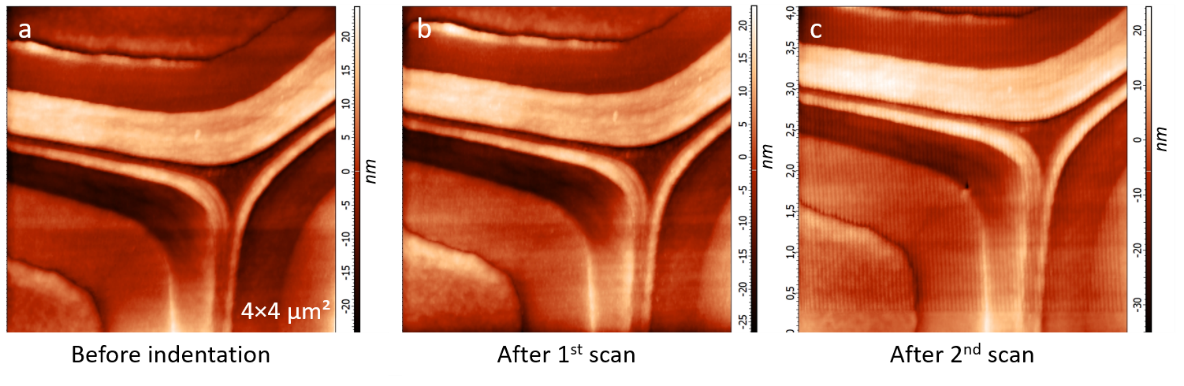


Supplementary figure S3: Topographic images acquired by semi-contact AFM of tension wood cell walls. a. before indentation. b. after first mapping with lows indentation depth. c. after second mapping with higher indentation depth.

## **4- Determination of maximum resolution**

As mentioned in the main text, the viscoplasticity index is equal to zero for none of the conditions tested. Indentation tests performed using force volume mode on wood cell walls are therefore never perfectly elastic. Indeed, residual prints can be observed by acquiring the indented area’s topography (Supplementary Fig. S3).

Because indentation tests stress a large volume below and around the contact surface, and that traditional contact models (Hertz, JKR, DMT) as well as the Sneddon formula are based on the hypothesis of a flat, semi-infinite indented medium, prints from the precedent indentation can impact the following indentations and resulting data^3,4^.

By following recommendations established for Berkovitch indenters^5^ and from the study of prints left after indentations, we define an indicator of the maximum resolution depending on the indentation depth and the tip used as follows:

$Maximum Resolution=3r$ , with r the average contact radius: $r=\frac{\surd(A(h_{c}))}{\pi}$.

We therefore wish to minimize the indentation depth, while respecting the minimum indentation criterion previously determined and allowing a correct modulus determination. In our case the maximum resolution is about 45 nm for PPP probe and 210 nm for DT probe.

## **5- Typical Force-indentation depth curves for several layers of tension wood**


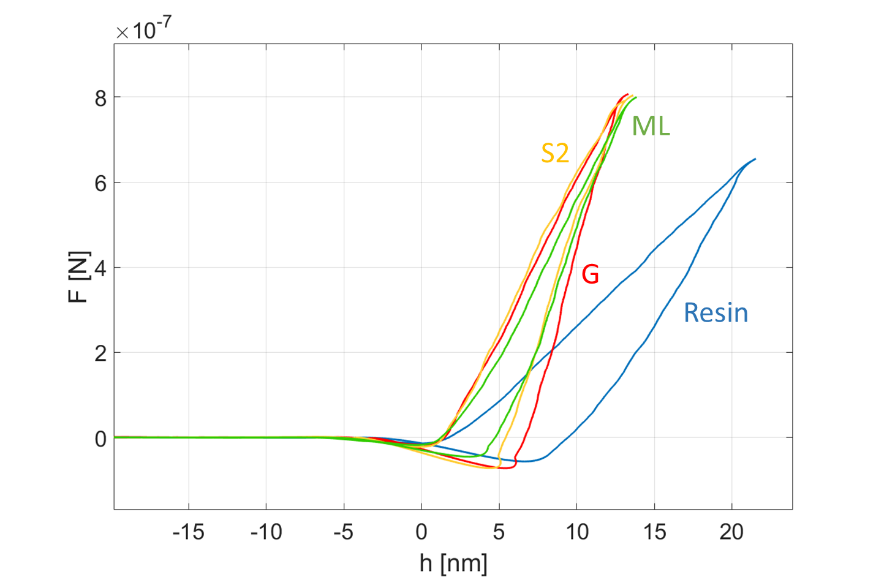


Supplementary figure S4: Force-indentation depth curves obtained in resin (blue), middle lamella cell corner (green), secondary layer (yellow) and G layer (red) using PPP tip.

## **6- Choice of polynomial degree for slope computation**

S is computed as the derivative of the polynomial fit at the beginning of the unloading curve. In this case, the values of S are computed using polynomials of degrees ranging from 1 to 8, each point corresponding to an average of 16 curves. The resulting values of S vary drastically from 0.07 to 0.12 µN/nm (Supp, Fig. S5a and S5b). Degrees 1 to 4 lead to an underestimation of the slope value as a result of a poor fit quality. Degrees 6 to 8 present high standard deviation because the fit becomes more and more dependant on local noise measurement.


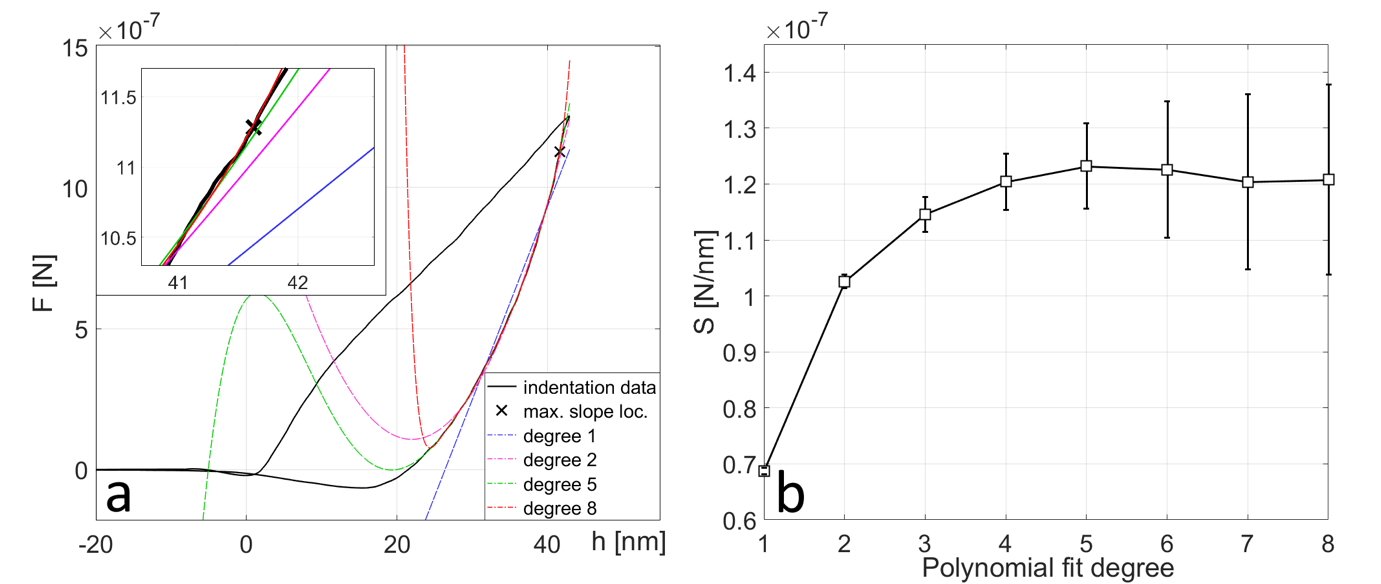


Supplementary figure S5: a. Indentation curve fitted with degree 1,2,5,8 polynomials, b. influence of polynom degree on the determination of slope S (n = 16).

## **7. Choice of the tip shape parameter Ɛ**

The value of Ɛ is linked to the indenter’s geometry: Ɛ = 0.72 for a conical tip, Ɛ =0.75 for a parabola of revolution, Ɛ = 1 for a cylindrical flat punch^1,2^. The parabola of revolution being the geometry closest to the AFM points, the value of 0.75 was chosen.

However, it has been highlighted that this coefficient has little influence on the final determination of the modulus (Supp, Fig. S6a). Thus, using extreme values such as 0.5 or 1 result in a relative deviation of less than 10% (Supp, Fig. S6b) for the extraction of the indentation modulus. The treatment carried out for Ɛ = 0 corresponds to a non-existent surface deflection near the contact.


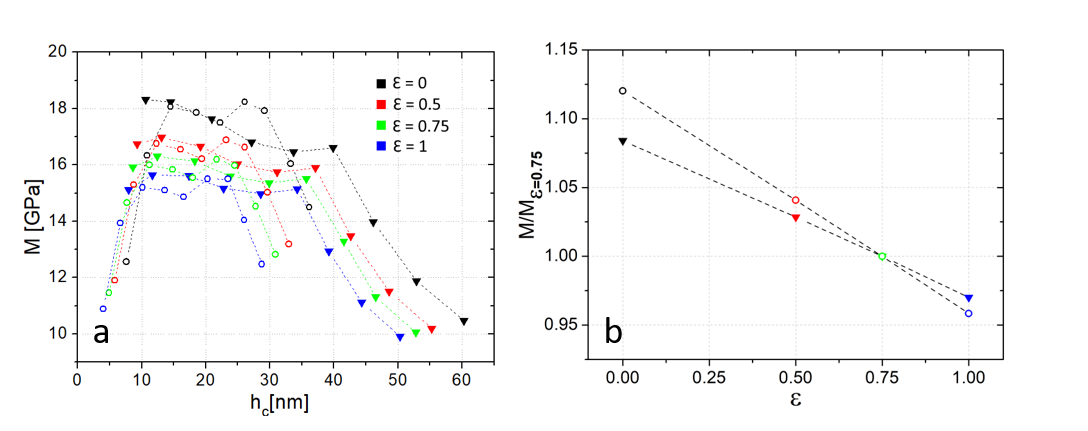


Supplementary figure S6: Influence of Ɛ on the indentation modulus’ determination. a. G layer’s indentation modulus as a function of indentation depth processed with different value of Ɛ with PPP (triangles) and DT (circles) probes. b. Average value of the indentation modulus as a function of Ɛ, normalized by the value for Ɛ = 0.75.

## **8- Example of sets of indentation measurements in the S2 layer**


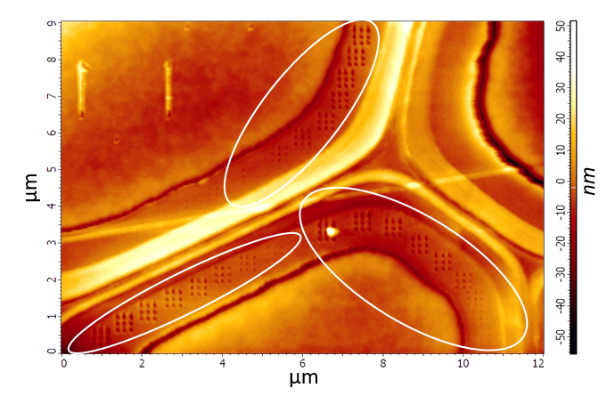


Supplementary figure S7: Topographic image acquired by AFM in tapping mode of tension wood cell walls after being indented. Prints of 3 series of indentation vs depth can be seen in enclosed areas.

**References**

1. VanLandingham, M. R., Villarrubia, J. S., Guthrie, W. F. & Meyers, G. F. Nanoindentation of polymers: An overview. *Macromol. Symp.* **167**, 15–43 (2001).

2. Oliver, W. C. & Pharr, G. M. An improved technique for determining hardness and elastic modulus using load and displacement sensing indentation experiments. *J. Mater. Res.* **7**, 1564–1583 (1992).

3. Wagner, L., Bader, T. K. & De Borst, K. Nanoindentation of wood cell walls: Effects of sample preparation and indentation protocol. *J. Mater. Sci.* **49**, 94–102 (2014).

4. Jakes, J. E., Frihart, C. R., Beecher, J. F., Moon, R. J. & Stone, D. S. Experimental method to account for structural compliance in nanoindentation measurements. *J. Mater. Res.* **23**, 1113–1127 (2008).

5. Sudharshan Phani, P. & Oliver, W. C. A critical assessment of the effect of indentation spacing on the measurement of hardness and modulus using instrumented indentation testing. *Mater. Des.* **164**, 107563 (2019).
